# Exploring the HIFs, buts and maybes of hypoxia signalling in disease: lessons from zebrafish models

**DOI:** 10.1242/dmm.021865

**Published:** 2015-11-01

**Authors:** Philip M. Elks, Stephen A. Renshaw, Annemarie H. Meijer, Sarah R. Walmsley, Fredericus J. van Eeden

**Affiliations:** 1 Department of Infection and Immunity, Medical School, The University of Sheffield, Sheffield, S10 2RX, UK; 2 The Bateson Centre, The University of Sheffield, Sheffield, S10 2TN, UK; 3 Institute of Biology, Leiden University, 2333 CC Leiden, The Netherlands; 4 MRC Centre for Inflammation Research, University of Edinburgh, Edinburgh, EH16 4TJ, UK

**Keywords:** Disease models, Hypoxia, Hypoxia-inducible factor, Zebrafish

## Abstract

A low level of tissue oxygen (hypoxia) is a physiological feature of a wide range of diseases, from cancer to infection. Cellular hypoxia is sensed by oxygen-sensitive hydroxylase enzymes, which regulate the protein stability of hypoxia-inducible factor α (HIF-α) transcription factors. When stabilised, HIF-α binds with its cofactors to HIF-responsive elements (HREs) in the promoters of target genes to coordinate a wide-ranging transcriptional programme in response to the hypoxic environment. This year marks the 20th anniversary of the discovery of the HIF-1α transcription factor, and in recent years the HIF-mediated hypoxia response is being increasingly recognised as an important process in determining the outcome of diseases such as cancer, inflammatory disease and bacterial infections. Animal models have shed light on the roles of HIF in disease and have uncovered intricate control mechanisms that involve multiple cell types, observations that might have been missed in simpler *in vitro* systems. These findings highlight the need for new whole-organism models of disease to elucidate these complex regulatory mechanisms. In this Review, we discuss recent advances in our understanding of hypoxia and HIFs in disease that have emerged from studies of zebrafish disease models. Findings from such models identify HIF as an integral player in the disease processes. They also highlight HIF pathway components and their targets as potential therapeutic targets against conditions that range from cancers to infectious disease.

## Abstract (precis)

**Summary:** Hypoxia signalling, mediated by HIF, is a crucial pathway in many disease processes. Here, we review current knowledge of HIF signalling and disease, focusing on recent findings from zebrafish models.

## Introduction

Cellular and tissue hypoxia are an everyday occurrence that cells must respond to rapidly in order to avoid metabolic shutdown and consequent death. All mammals control the cellular response to low oxygen levels through regulation of the hypoxia-inducible factor α (HIF-α) transcription factor family (members of which are discussed in more detail below). Unlike many transcription factors, the levels of HIF-α are primarily controlled post-translationally. HIF-α protein activity is controlled by two families of oxygen-sensing hydroxylases: prolyl hydroxylase domain-containing proteins (PHDs) and factor inhibiting HIF (FIH) (Epstein et al., 2001; Lando et al., 2002). In normoxia, PHDs hydroxylate HIF-α and target it for proteasomal degradation, which is facilitated by the binding of von Hippel-Lindau tumour suppressor (pVHL) protein. In hypoxia, PHD enzyme activity is reduced, owing to the oxygen requirement of the hydroxylase activity, and HIF-α is stabilised (Huang et al., 1998; Kallio et al., 1999). Once stabilised, the HIF-α subunit forms a nuclear heterodimeric complex with its constitutively stable counterpart HIF-β [or aryl hydrocarbon nuclear translocator (ARNT)] to transcribe target genes that contain a HIF-responsive element (HRE) in their regulatory regions (Huang et al., 1996; Kaelin and Ratcliffe, 2008; Semenza and Wang, 1992; Wenger, 2002).

This year, 2015, marks the 20th anniversary of the identification of HIF-1α as the protein responsible for the cellular response to hypoxia, and since its discovery it has been implicated in disease (Wang and Semenza, 1995). Oxygen levels vary greatly in health, and there are significant oxygen gradients across tissues during homeostasis (Lokmic et al., 2012). These oxygen gradients are disrupted in a range of diseases. Modulation of HIF-α has been mechanistically linked to the progression and severity of a number of disease processes, including cancer and inflammatory diseases (Semenza, 2012). Over the last 20 years, major leaps in our understanding of hypoxia and HIF signalling have emerged from *in vitro* cell-culture studies, which offer powerful tools for investigating hypoxia and the HIF pathway (Bruick and McKnight, 2001; Chan et al., 2005; Salceda and Caro, 1997). In disease, tissue hypoxia is generated in a complex tissue environment, with wide variation in the local levels of oxygen because of differences in oxygen supply and consumption. In the last decade, *in vivo* models have complemented cell-line studies, giving a more physiologically relevant setting in which to understand the interrelationship of hypoxia and disease. The most widely used animal models to understand hypoxia and HIF are rodents. Mice and rats are highly amenable to manipulation and are small enough to fit into hypoxic chambers for long periods of time (Yu and Hales, 2011). The development of Cre-*lox* conditional knockout systems in mice has allowed cell- and tissue-specific HIF-1α and HIF-2α knockout models to be created that have been instrumental in our understanding of the roles of hypoxia and HIF in specific cell types and tissues (Cramer et al., 2003; Kapitsinou et al., 2014; Schipani et al., 2001).

HIF is a major regulator of homeostasis and has wide-ranging effects: from the cellular level to a local level (for example, in a tumour) to systemic effects across the entire organism. The zebrafish (*Danio rerio*) is a genetically tractable organism that has recently come to light as a useful model for studying hypoxia and HIF in disease. A major advantage of zebrafish is that they have optically transparent larvae, providing an unprecedented opportunity to visualise disease processes *in vivo* using fluorescence microscopy, from holistic whole-body phenotypes to individual cell behaviour. Other advantages of the zebrafish system include medium-to-high throughput drug screening (via addition of small-molecule compounds to the embryo water; Robertson et al., 2014a) and genetic tractability [especially with recently improved genome-editing technology via CRISPR (clustered regularly interspaced short palindromic repeats)/Cas9 (CRISPR associated protein 9) technology (see Box 1); Hruscha et al., 2013; Varshney et al., 2015]. Zebrafish have conserved homologues of most human genes and have all the pathway components of HIF signalling. Initially employed as a model of developmental biology, in the last 15 years zebrafish research has extended to include disease modelling, and there are now numerous diseases modelled in the zebrafish, from tuberculosis to Parkinson's disease (Flinn et al., 2008; Renshaw and Trede, 2012; Torraca et al., 2014).

The mechanisms of hypoxia and HIF stabilisation must be elucidated further in the context of *in vivo* disease models to identify successful avenues for drug discovery and development against disease. In this Review, we outline the zebrafish models that are available for investigating hypoxia and HIF in disease settings. We discuss the conservation of hypoxia-signalling components, followed by the methods employed to manipulate hypoxia signalling in live zebrafish models. In the second part of the Review, we discuss recent advances in hypoxia and disease that have emerged from zebrafish studies, as well as the challenges facing this research field. We also assess how zebrafish can be used to further our understanding of HIF signalling and disease.

## Hypoxia signalling in zebrafish

There are three mammalian isoforms of HIF-α, namely HIF-1α, HIF-2α and HIF-3α (Ema et al., 1997; Gu et al., 1998; Tian et al., 1997; Wang and Semenza, 1995). The assembly of the HIF transcription factor in response to low levels of oxygen depends on the accumulation of the HIF-α subunit (Kaelin and Ratcliffe, 2008). Although many transcription factors are regulated at a transcriptional level, requiring *de novo* protein synthesis, HIF-α is primarily regulated post-translationally to allow for a rapid response to decreasing oxygen levels (Berra et al., 2001; Moroz et al., 2009; Wang et al., 1995). HIF biology is well conserved across vertebrates, with all having at least three HIF-α subtypes, HIF-1α, HIF-2α and HIF-3α (Hampton-Smith and Peet, 2009). Of these, HIF-1α and HIF-2α are the best characterised across species and are the most highly expressed across tissues (Prabhakar and Semenza, 2012). The role of HIF-3α is not yet clear, with multiple splice variants having opposing effects on HIF-1α and HIF-2α signalling, some acting as promoters and others as inhibitors (Maynard et al., 2007, 2003; Zhang et al., 2014).

**Fig. 1.**
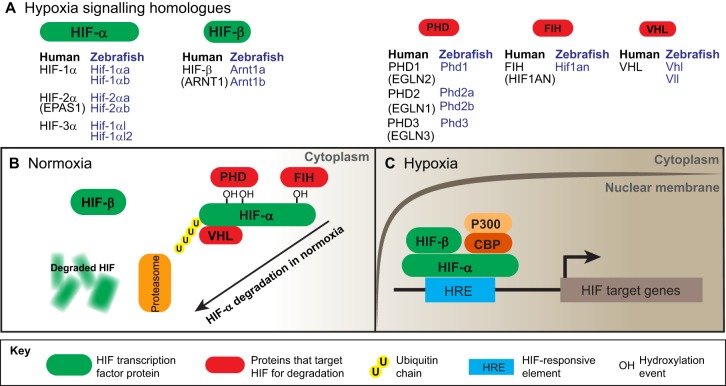
**Schematic diagram of the HIF-signalling system and zebrafish homologues.** (A) Proteins of the HIF-signalling pathway are illustrated, with HIF proteins in green and proteins that target HIF for degradation in red. The human isoforms are listed, with their zebrafish counterparts in blue text. (B) In normoxia, the hydroxylase enzyme PHD and the VHL protein target HIF-α for ubiquitination in the cytoplasm and subsequent degradation by the proteasome. A second hydroxylase enzyme, FIH, hydroxylates a C-terminal asparagine residue on HIF-α in normoxia to repress the transactivation function of HIF. (C) In hypoxia, hydroxylase enzymes are silenced, and HIF translocates to the nucleus and binds its cofactors, where it upregulates target gene expression by binding to HREs in their regulatory regions. Abbreviations: CBP, CREB-binding protein; FIH, factor inhibiting HIF; HIF, hypoxia-inducible factor; HREs, HIF-responsive elements; P300, E1A binding protein; PHD, prolyl hydroxylase domain-containing proteins; VHL, von Hippel-Lindau.

Zebrafish homologues of HIF-α are referred to as Hif-1αa, Hif-1αb, Hif-2αa, Hif-2αb, Hif-3αa and Hif-3αb (Fig. 1 and Table 1). The a and b forms have arisen from a genome duplication event in the teleost lineage, more than 100 million years ago (Postlethwait et al., 2000; Rojas et al., 2007; Rytkonen et al., 2013, 2014). Sequence homology shows that the zebrafish Hif-1αb variant is more closely related to human HIF-1α than to zebrafish Hif-1αa, with an amino acid identity of 57.8 and 44.1%, respectively (Rytkonen et al., 2014). Furthermore, Hif-1αa and b forms are differentially expressed, with the Hif-1αb homologue being more highly expressed than Hif-1αa when mRNA levels are assessed by *in situ* hybridisation (Elks et al., 2011; Rojas et al., 2007). Hif-1αa lacks one of the regulatory LXXLAP hydroxylation sites, whereas Hif-1αb retains both LXXLAP domains (Elks et al., 2011). Functional expression and overexpression data are consistent with Hif-1αb being the key zebrafish homologue in the hypoxic response (Elks et al., 2011; Kopp et al., 2011). Zebrafish Hif-2αa and Hif-2αb are more closely related to each other than to their Hif-1α equivalents, both in terms of transcript expression levels and amino acid identity to human HIF-2α (56.1 and 53.7% amino acid identity, respectively; Rytkonen et al., 2014). Both have the two regulatory LXXLAP hydroxylation sites. Although Hif-2αa is more widely studied in zebrafish (Thompson et al., 2014), there are no data to suggest that Hif-2αb cannot play an active role in zebrafish hypoxia signalling. Similar to in mammalian systems, Hif-3α has multiple splice variants in the zebrafish and is not as widely studied as Hif-1α and Hif-2α (Zhang et al., 2014). Like Hif-1α and Hif-2α, Hif-3α is duplicated in the zebrafish, described as Hif-3αa and Hif-3αb (Rytkonen et al., 2014). A recent antibody study indicates that all Hif-α proteins are detectable in the zebrafish embryo from 1 day postfertilisation and suggests that Hif-1αb is the most upregulated protein in the embryo response to hypoxia (Koblitz et al., 2015).

**Table 1.**
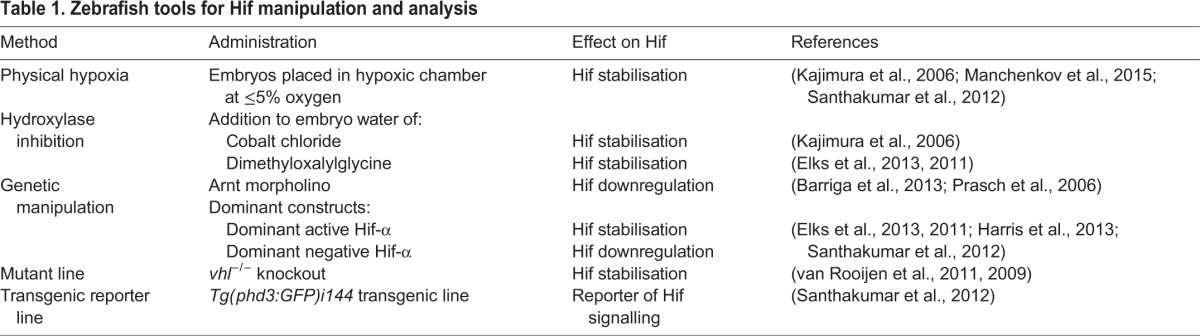
**Zebrafish tools for Hif manipulation and analysis**

In normoxia, PHD enzymes hydroxylate HIF-α on two conserved prolyl residues in the LXXLAP hydroxylation sites, triggering binding and ubiquitination by pVHL, leading to the degradation of HIF-α by the proteasome (Huang et al., 1998; Kallio et al., 1999; Salceda and Caro, 1997). HIF-α degradation, via hydroxylation by PHD enzymes, is the major regulatory mechanism for controlling the HIF response. However, HIF signalling is also fine-tuned by transcriptional regulation of HIF-α and by blocking its transcription factor activity by a second HIF-α regulatory hydroxylase family protein, FIH (Lando et al., 2002). The human genome contains three functional PHD enzyme family members, namely PHD-1 (EGLN2), PHD-2 (EGLN1) and PHD-3 (EGLN3). Zebrafish have one homologue of each, apart from PHD-2, which is duplicated: Phd-1 (Egln2), Phd-2a (Egln1a), Phd-2b (Egln1b) and Phd-3 (Egln3). The functional activity and expression of Phd enzymes are not widely studied in zebrafish. However, Phd-3 is the most highly upregulated variant when Hif-α is stabilised in a Vhl knockout zebrafish line, demonstrating the same negative feedback loop observed in mammals (Santhakumar et al., 2012; van Rooijen et al., 2011). Zebrafish have a single homologue of VHL (Vhl), and a less well-characterised VHL-like gene (*vll*) (Metelo et al., 2015; van Rooijen et al., 2011, 2010, 2009). Zebrafish Vhl shares 52% amino acid identity with human VHL, and knockout studies indicate that it is functional in the hypoxic response (van Rooijen et al., 2010). Zebrafish have one functional homologue of FIH (Fih) that is highly conserved with the human protein, with 79% amino acid identity. The function of Fih in negatively regulating Hif has yet to be confirmed in the zebrafish, but functional conservation is suggested at the protein structure level, with the enzymatically active Jumonji (JmjC) domain having 96% homology to that in human FIH (So et al., 2014). Furthermore, the transcript expression of *fih-1* is comparable to that of *vhl* and *hif-1αb* at later stages of development (25 and 36 h postfertilisation), indicating a functional role in hypoxia signalling (So et al., 2014).

In hypoxia, the hydroxylases are silenced, which results in the stabilisation of HIF-α. Knockdown studies have demonstrated that, as in humans, zebrafish Hif-α must bind to its partner, Arnt, as a heteromer to signal (Elks et al., 2011; Prasch et al., 2006). Zebrafish have two Arnt1 homologues, Arnt1a and Arnt1b, with the shorter Arnt1a form seemingly non-functional in *in vitro* studies (Prasch et al., 2006). The conservation of the HIF-signalling components in the zebrafish extends to the level of HIF-responsive elements (HREs) in promoters of known HIF targets, including Phd-3 and insulin-like growth factor binding protein-1, among others (Egg et al., 2013; Greenald et al., 2015; Kajimura et al., 2006; Kulkarni et al., 2010; Santhakumar et al., 2012).

## Manipulation of hypoxia signalling in zebrafish

**Fig. 2.**
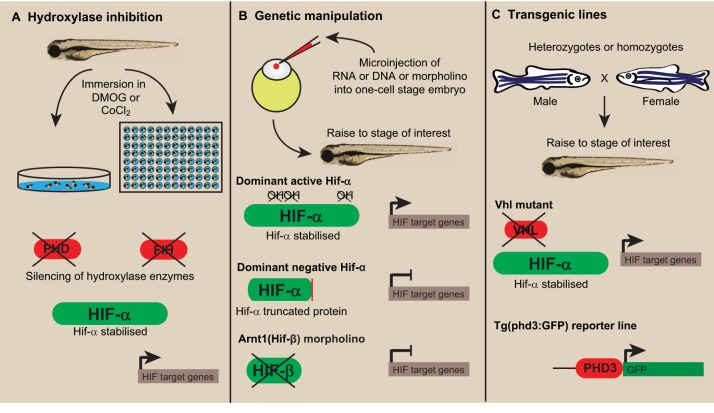
**Manipulation of hypoxia signalling in zebrafish.** (A) The zebrafish is amenable to *in vivo* pharmacological manipulation, which enables evaluation of the effects of Hif-signalling modulation and any resultant disease-related processes in an intact organism. Drugs such as DMOG and cobalt chloride can be added directly to the embryo water to inhibit the hydroxylase enzymes Phd and Fih, thus allowing the expression of Hif target genes in normoxia. Zebrafish embryos can be used for testing many drugs at a time because individual embryos can fit into a 96-well plate format. (B) Hif signalling can be manipulated genetically in zebrafish embryos by microinjection of RNA or DNA constructs or antisense oligonucleotide morpholinos targeting Hif-pathway components into the one-cell stage. The injection of dominant active Hif-α constructs can be used to stabilise Hif-α, because in these constructs the regulatory proline targets of Phd and the asparagine target of Fih are mutated into non-hydroxylatable amino acids. Dominant negative Hif-α variants, instead, contain truncations that remove the transactivation domains required for signal transduction. In addition, morpholinos against the Hif-1β/Arnt subunits can successfully knock down Hif signalling. (C) Transgenic lines are available to manipulate and follow Hif signalling *in vivo*. Notably, the *vhl^−/−^* mutants can upregulate Hif signalling via Hif-α stabilisation, whereas the *Tg(phd3:GFP)i144* transgenic line can be used as a reporter of Hif activity. Abbreviations: Arnt, aryl hydrocarbon nuclear translocator; DMOG, dimethyloxalylglycine; Fih, factor inhibiting HIF; GFP, green fluorescent protein; HIF, hypoxia-inducible factor; Phd, prolyl hydroxylase domain-containing proteins; *phd3*, prolyl hydroxylase domain-containing protein-3; *vhl*, von Hippel-Lindau.

The genetic conservation of HIF-signalling components across vertebrates means that *in vivo* studies in simpler vertebrates, such as in murine models and zebrafish, have relevance to human hypoxia signalling. Zebrafish are highly amenable to pharmacological and genetic manipulation, and these properties have led to the creation of a number of methods to modulate Hif signalling *in vivo* (Fig. 2). The benefits of modulating Hif in the zebrafish include being able to follow the effects and any resulting disease-related processes in an intact organism, from the whole-body response down to the level of individual cells and cell types.

### Physical hypoxia

The classical cellular models employed to investigate HIF signalling use physical hypoxia. In cell-line and tissue-culture systems, this relatively simple procedure can be performed in a hypoxic chamber. However, hypoxia is not simple to achieve when using *in vivo* murine models because of their large size and the need to feed and care for the animals while in the chamber (Hancher and Smith, 1975). Zebrafish embryos are much smaller than mice (2-5 mm across) and do not need to feed until after 5 days postfertilisation. They can therefore be treated much like a cell-culture model and can be left undisturbed in a hypoxic chamber for prolonged periods of time (Manchenkov et al., 2015; Santhakumar et al., 2012). Zebrafish embryos are relatively tolerant to low oxygen, but care should be taken to balance the carbon dioxide levels to ensure that pH remains neutral. Any media used should also be pre-incubated in low oxygen before their addition to the embryos to ensure that low oxygen levels are maintained. A level of 5% hypoxia is sufficient to activate Hif signalling in the zebrafish, and this level has been used to demonstrate that downstream Hif-α targets are conserved (Kajimura et al., 2006; Santhakumar et al., 2012). Brief incubation periods have demonstrated that zebrafish embryos are amenable to hypoxic preconditioning that is protective against later hypoxic events, a phenomenon observed in mammals that is not currently understood (Manchenkov et al., 2015).

### Pharmacological inhibition of oxygen-sensing hydroxylases

The treatment of zebrafish with small molecular pharmaceuticals is a powerful means by which to manipulate genetic pathways and enzyme activity *in vivo*. The liquid environment of zebrafish larvae facilitates the temporal manipulation of pathways through the addition of drugs direct to the embryo media. Drugs can also be added and washed off repeatedly. The small size of zebrafish embryos allows medium- to high-throughput drug screening in a 96-well plate format, making zebrafish a powerful screening model (Kaufman et al., 2009; Robertson et al., 2014a). The best-characterised pharmaceuticals used to manipulate HIF-α are pan-hydroxylase inhibitors that inhibit PHD and FIH hydroxylase family members to stabilise HIF-α (Robinson et al., 2008). The hydroxylase inhibitors cobalt chloride (CoCl_2_) and dimethyloxalylglycine (DMOG) have been used in the zebrafish systems to control Hif-α stabilisation temporally (Elks et al., 2013, 2011; Kajimura et al., 2006; Fig. 2A). However, CoCl_2_ has to be used at high concentrations (10 mM) to achieve hydroxylase inhibition and has been shown to have off-target toxic effects in fish (Saeedi Saravi et al., 2009). DMOG is a more specific 2-oxalylglycine hydroxylase inhibitor and is less toxic than CoCl_2_. Nonetheless, it is important to consider that, like CoCl_2_, DMOG is a pan-hydroxylase inhibitor and will have effects on biological processes independently of Hif. Both treatments stabilise Hif-α in the zebrafish model and induce downstream target gene expression (e.g. transcription of *phd3*; Elks et al., 2011; Kajimura et al., 2006).

### Genetic manipulation and visualisation of Hif-α signalling

Targeting Hif-α genetically is a powerful and specific approach to studying the roles of Hif signalling in disease. Morpholinos (blocking antisense oligonucleotides) against Hif-1αb and Arnt subunits have been successfully used in zebrafish embryo models to knock down Hif signalling (Barriga et al., 2013; Prasch et al., 2006; Fig. 2B). Morpholinos are limited by off-target, non-specific events, estimated to occur in up to 50% of morpholinos (Kok et al., 2015). To circumvent these off-target effects, dominant active and dominant negative *hif-α* constructs have been created to manipulate Hif signalling *in vivo* (Elks et al., 2011; Fig. 2B). These constructs are based on manipulation of HIF-α in human cell-line models (Chan et al., 2005; Linke et al., 2004; Manotham et al., 2005). Dominant negative Hif-α variants contain truncations (of ∼330 amino acids) that remove the N-terminal and C-terminal transactivation domains (N-TAD and C-TAD) required for signal transduction. They downregulate Hif signalling by binding to cofactors (such as Hif-1β), but being unable to transduce a signal. Dominant active Hif-α variants are inherently stable, even in normoxia, because the regulatory proline targets of Phd have been mutated into non-hydroxylatable amino acids (along with the asparagine target of Fih, which lifts the transcriptional block mediated by Fih). Dominant constructs can be expressed transiently over all tissues of the embryo through the injection of synthesised RNA at the one-cell stage without any overt signs of off-target expression or toxicity (Elks et al., 2011, 2013; Harris et al., 2013). A major advantage of modulating Hif-α using dominant variants is that they can be driven in specific tissues of interest, using tissue- and cell-type-specific promoters or the GAL4/UAS (yeast transcription activator protein/upstream activation sequence) system (Elks et al., 2011, 2013, 2014; Jopling et al., 2012).

Efficient knockout technology was developed in the zebrafish in the early 2000s, based on random mutagenesis followed by high-throughput sequencing (TILLING; Wienholds et al., 2003). Mutant alleles for *vhl* were generated as one of the first knockouts produced by TILLING and have proved to be a tractable model for studying the overactivation of Hif signalling in zebrafish (van Rooijen et al., 2009).

Transgenic fluorescent lines are important tools that are used in zebrafish disease models to investigate biological processes in different tissue systems, in real time. Santhakumar et al. (2012) took advantage of transgenic technology to make a hypoxia-signalling reporter zebrafish line. From gene-expression profiling studies of the *vhl^−/−^* zebrafish mutant, it was noted that *phd3* is highly upregulated in a consistent fashion compared with wild-type and heterozygous siblings (van Rooijen et al., 2011). By driving green fluorescent protein (GFP) expression with the *phd3* promoter, the *Tg(phd3:GFP)i144* transgenic line provides a read out for hypoxia and Hif signalling *in vivo* (Santhakumar et al., 2012). This reporter line thus allows the functional stabilisation of Hif-α to be imaged in real time in zebrafish embryos and is an important tool for investigating the activation of Hif signalling *in vivo*.

Recent advances in genome-editing technology, including TALEN (transcription activator-like effector nucleases) and CRISPR/Cas9 systems (see Box 1), are now enabling the efficient generation of specific gene knockouts in zebrafish, which are highly amenable to these technologies (Clark et al., 2011; Hruscha et al., 2013; Varshney et al., 2015). We are entering an exciting age of genome editing *in vivo*; the ability to generate new zebrafish Hif-signalling mutants using the TALEN and CRISPR genome-editing technologies is a tantalising future prospect.

**Box 1. Glossary**

**Chronic obstructive pulmonary disease (COPD):** a condition characterised by chronic inflammation of the lung airways and alveoli.

**Clustered regularly interspaced short palindromic repeats (CRISPR/Cas9):** a genome-editing technology that enables targeted disruption of the coding sequence of a target gene(s) using an appropriate guide RNA to direct a Cas9 nuclease to a specific genome location.

**Erythropoietin:** a glycoprotein hormone that controls erythropoiesis (red blood cell formation).

**Intracellular iron regulatory protein 1 (IRP1):** a protein involved in the control of iron metabolism and in catalysing the conversion of citrate to isocitrate.

**Liposome**: a spherical vesicle having at least one lipid bilayer that can be used as a vehicle for administration of drugs.

**Mammalian target of rapamycin (mTOR):** a serine/threonine protein kinase that regulates many cellular processes, including cell growth, proliferation, motility, survival, protein synthesis, autophagy and transcription.

**Nitric oxide synthase (NOS):** an enzyme that catalyses the production of nitric oxide, an important cellular signalling molecule involved in wide-ranging physiological responses, including angiogenesis, neurotransmission and immune defence.

**Nuclear factor κ-light-chain-enhancer of activated B cells (NFκB):** a protein complex that plays a key role in regulating the immune response to infection by regulating the production of cytokines.

**Phosphoinositide 3-kinase (PI3K):** a family of related intracellular signal transducer enzymes that phosphorylate the 3-position hydroxyl group of the inositol ring of phosphatidylinositol, having wide-ranging cellular effects, including cell growth, proliferation, differentiation, motility, survival and intracellular trafficking.

**Polycythaemia**: a condition associated with a high concentration of red blood cells in the blood.

**Polymersomes**: artificial vesicles that can contain and deliver drugs.

**Single plane illumination microscopy (SPIM):** a microscopy method that employs a sheet of laser light to illuminate the sample.

**Transcription activator-like effector nucleases (TALENs):** a genome-editing technology that enables targeted disruption of the coding sequence of a target gene(s) using an appropriate guide RNA to direct a Cas9 nuclease to a specific genome location.

## Hypoxia signalling in disease: *in vivo* insights from zebrafish

**Fig. 3.**
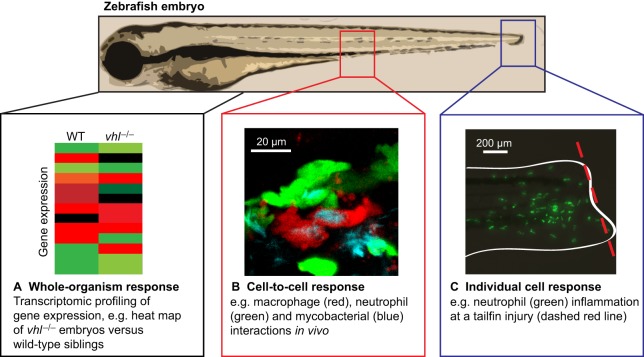
**Zebrafish as a model to investigate the role of Hif in disease.** (A) Transcriptional profiling in zebrafish *vhl^−/−^* mutant embryos has been used to observe the response to overactivation of Hif signalling at the whole-organism level. (B) Complex cell type-cell type interactions can be studied in situations where Hif is activated, taking advantage of fluorescent transgenic lines that label specific groups of cells, e.g. in macrophage, neutrophil and bacterial interactions. (C) Zebrafish can also be used to investigate individual cell behaviours, such as the behaviour of neutrophils at a site of inflammation (e.g. tailfin transection).

Hypoxia and HIF signalling play crucial roles in the progression of a wide range of diseases (Semenza, 2014). The complexity of HIF activation during *in vivo* disease processes means that this process is difficult to model successfully in cell- and tissue-culture assays. For example, the hypoxic centres of cancerous tumours or tuberculosis granulomas are not situations that can be modelled easily *ex vivo*. The zebrafish has been adopted as a simple whole-vertebrate model to investigate disease systems, complementing cell and murine models in the quest to understand the roles of hypoxia in disease (Fig. 3). Here, we discuss recent insights to have emerged from studies in zebrafish disease models.

### Hypoxia signalling in cancer and angiogenesis

An important HIF-α target is vascular endothelial growth factor (VEGF; Forsythe et al., 1996; Shweiki et al., 1992), a master regulator of angiogenesis (Leung et al., 1989). Historically, this mechanistic link has been drawn from the roles of hypoxia in tumour vascularisation (Connolly et al., 1989). Tissue hypoxia is a crucial component of the tumour microenvironment and becomes evident as a tumour undergoes rapid uncontrolled growth, causing it to outgrow the local circulation and leading to its centre becoming hypoxic. Localised HIF-α expression drives expression of VEGF to increase blood circulation to the centre of the tumour, supporting further rapid growth. However, excessive VEGF leads to aberrant vessel formation, leading to vessels that are leaky and inefficient and that promote the escape of cancer cells and subsequent metastases (Welti et al., 2013). This has been shown to be true in a zebrafish model where physical hypoxia increases the dissemination and invasion of a mouse fibrosarcoma implanted into zebrafish embryos, with both dissemination and invasion being correlated with increased VEGF and tumour vascularisation (Lee et al., 2009). Nonetheless, this model is not without its limitations because hypoxia is exerted across the whole organism, rather than being the localised hypoxia that is observed in cancerous tumours. The development of the *Tg(phd3:GFP)i144* line has enabled the visualisation of localised hypoxic signalling in tumours in *vhl*^−/−^ knockout zebrafish at physiological oxygen levels (Santhakumar et al., 2012). This study was performed in adults, but by using a combination of transgenic and imaging technologies in embryos, zebrafish models of tumour hypoxia have the potential to become an important model for screening therapeutics for cancer studies that has yet to be exploited. The pharmacological targeting of HIF has exciting potential as an anti-tumour therapy to limit tumour growth and decrease metastases. However, this prospect is complicated by tumour cells adapting to the inhibition of HIF through metabolic reprogramming, potentially aiding cancer cell survival, making this treatment strategy something of a double-edged sword (Golinska et al., 2011).

The ease of imaging intersegmental vessels has made the zebrafish a valuable model for studying angiogenesis, both in development and in cancer studies (Lawson and Weinstein, 2002). Intersegmental vessels are blood vessels that run between the somite muscle blocks of the embryonic zebrafish connecting the major vessels at the dorsal and ventral sides of the trunk. The direct transcriptional regulation of VEGF by HIF, as well characterised in mammals, has been demonstrated in zebrafish (Maeda et al., 2008). Using bright fluorescent transgenic lines that mark vessel endothelia [e.g. the *Tg(fli:GFP)* transgenic line], it has been shown that the potentially clinically relevant anti-angiogenic properties of methyl *tert*-butyl ether, with a previously unknown mechanism of action, are a result of the downregulation of Hif- and Vegf-dependent angiogenesis (Bonventre et al., 2013). More recently, the direct HIF-VEGF link has been investigated and applied to other disease situations that are not traditionally associated with angiogenesis. In a naturalised zebrafish model of tuberculosis (using *Mycobacterium marinum* infection), it has been demonstrated that inhibition of vascular invasion into developing granulomas reduces bacterial growth and dissemination within the host and might be a novel therapeutic approach to reduce tuberculosis infection in humans (Oehlers et al., 2015).

As well as the direct link between HIF and VEGF, the zebrafish model has identified more subtle regulatory mechanisms of angiogenesis in disease. The *vhl*^−/−^ mutant has been used as a convenient model of Hif-driven angiogenesis and has identified additional regulatory mechanisms of angiogenesis, which are independent of Vegf (van Rooijen et al., 2010, 2009). Using the *vhl*^−/−^ model, it was demonstrated that hypoxia-mediated angiogenesis differs from developmental angiogenesis in its need for the presence of blood flow (Watson et al., 2013). The validation of the *vhl*^−/−^ mutant as a robust model for hypoxia-driven angiogenesis has provided a clinically relevant model for vascular retinopathies (eye disorders caused by persistent or acute damage to the retina; van Rooijen et al., 2011, 2010). Apart from its role in HIF turnover, VHL is also a tumour suppressor, the loss of which causes VHL-associated polycythaemia disease (an increase in red blood cell count; see Box 1) in humans (Pastore et al., 2003). The *vhl*^−/−^ zebrafish mutant has been used as a tractable animal model for its Hif-dependent functions in the development of polycythaemia (van Rooijen et al., 2009).

### Hypoxia signalling in inflammation and tissue regeneration

Tissue hypoxia can occur following injury as blood vessels become damaged and blood oxygen delivery to the local region is restricted (Manresa et al., 2014). Tissue injury causes inflammation and the recruitment of immune cells into the wound to clear damaged cells and to protect against infection. Innate immune cells (leukocytes) are the first cells to respond to tissue injury or infection (Palazon et al., 2014) and must be able to sense local oxygen changes, rapidly adapting to these changing conditions to operate in areas of low oxygen tension. Inflammation is necessary for tissue repair, but must resolve in order for tissue regeneration and homeostasis. HIF signalling plays a crucial role in the regulation of inflammation, because it contributes to the regulation of the lifespan and immune activity of leukocytes (Hannah et al., 1995). Inflammation also acts to promote HIF via nuclear factor κ-light-chain-enhancer of activated B cells (NFκB; see Box 1), demonstrating the tight molecular link between signalling systems (Jung et al., 2003). The tissue damage observed in inflammatory diseases, such as in chronic obstructive pulmonary disease (COPD; Box 1), can be seen as a result of the failure of timely resolution of inflammation (Hallett et al., 2008; Walmsley et al., 2011). It was previously thought that removal of neutrophils during resolution of inflammation was exclusively regulated by the death of neutrophils by apoptosis (Haslett et al., 1994) and their subsequent clearance by macrophage efferocytosis. A zebrafish model of neutrophil inflammation using a sterile tailfin transection (Fig. 3C) has allowed the investigation of a new mechanism for neutrophil removal from inflammatory sites: the reverse migration of neutrophils (Elks et al., 2011; Renshaw et al., 2006). Although the idea of immune cells migrating away from wound sites has been identified in other vertebrate models, it has proved challenging to characterise definitively (Buckley et al., 2006). In the transparent zebrafish model, *in vivo* real-time observations have shown that reverse migration and apoptosis operate in parallel to the resolution of inflammation and can both be manipulated by changes in Hif signalling (Elks et al., 2011; Holmes et al., 2012; Mathias et al., 2006; Robertson et al., 2014a; Yoo and Huttenlocher, 2011). The stabilisation of Hif-α signalling in the zebrafish model, by DMOG or by genetic stabilisation of *hif-1αb* or *hif-2αa*, can delay the process of reverse migration, decrease neutrophil apoptosis and thereby delay the resolution of inflammation (Elks et al., 2011; Thompson et al., 2014). Importantly, the relevance of the zebrafish model to human disease was demonstrated by the replication of two naturally occurring gain-of-function human HIF-2α mutations in the zebrafish. G487R and G487W mutations in zebrafish Hif-2αa phenocopied a neutrophil apoptosis phenotype observed in people with the equivalent gain-of-function mutations (Thompson et al., 2014). The recognition of reverse migration as a potential anti-inflammatory approach has opened up new avenues of potential drug treatments for currently untreatable inflammatory diseases (Lucas et al., 2014). If the mechanisms of reverse migration could be identified, then targeting these mechanisms might offer an effective treatment to alleviate diseases, such as chronic obstructive pulmonary disease, by removing inflammation, rather than by treating the symptoms alone. Once inflammation has resolved, tissue regeneration can occur and homeostasis can be restored.

Hypoxia signalling is integrally involved in all stages of wound repair and regeneration (Nauta et al., 2014). The regenerative capacity of the zebrafish is much greater than that of mammals, making it an attractive vertebrate model of tissue regeneration after injury (Goessling and North, 2014). This is especially true at embryonic stages where the liver, heart, eye and fins are able to regenerate completely after injury, but is also retained into adulthood in some organs. The regeneration of zebrafish cardiomyocytes has been shown to be dependent partly on Hif-1α signalling in adult zebrafish, which can survive and regenerate an injury of up to 20% of the heart tissue, identifying Hif-1α as a potential drug target for regenerative medicine (Jopling et al., 2012; Parente et al., 2013).

### Hypoxia signalling in infection

Multidrug resistance is a worldwide problem in bacterial infections, including infections with *Mycobacterium tuberculosis* (the cause of tuberculosis) and *Staphylococcus aureus* (the cause of MRSA) (Anwar et al., 2009; Janbaz et al., 2012). As with inflammatory processes, HIF-1α has activating effects on leukocytes during infection (Peyssonnaux et al., 2005; Zarember and Malech, 2005). Hypoxia signalling is upregulated in cell-line and in murine macrophage models of bacterial infection, and HIF-1α overexpression is known to upregulate the antimicrobial activities of leukocytes, including phagocytosis, bacterial killing and leukocyte lifespan (Peyssonnaux et al., 2005; Walmsley et al., 2006; Zarember and Malech, 2005). Intriguingly, upregulation of HIF-α in infection can be independent of a decrease in oxygen tension (Palazon et al., 2014). HIF-α has therefore become increasingly investigated as a druggable target against bacterial infections, a strategy that would, in theory, be effective against multidrug-resistant infections because it targets host, not pathogen, biology.

**Fig. 4.**
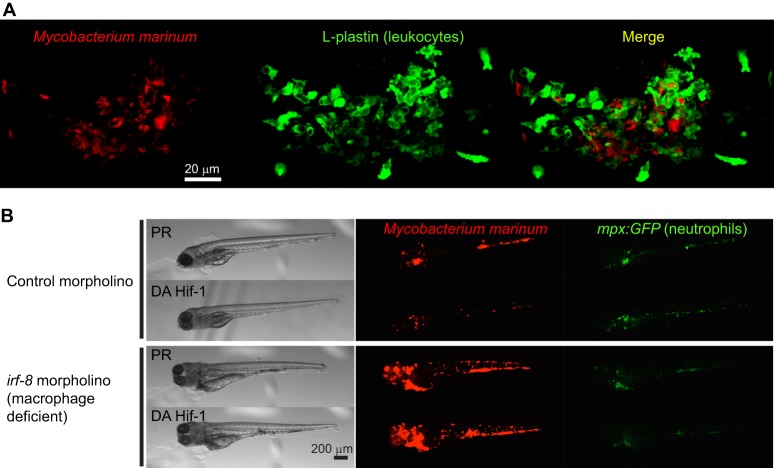
**Hif-1α stabilisation reduces bacterial burden in zebrafish embryos.** (A) Zebrafish embryos were infected with *Mycobacterium marinum* (red) at 1 day postfertilisation by injection into the caudal vein. By 4 days postinfection, foci of *M. marinum* (red) are surrounded by infected and uninfected leukocytes (green; L-plastin antibody staining) in structures known as granulomas. (B) Stabilisation of Hif-1α, using dominant active (DA) Hif-1αb reduces the bacterial burden of zebrafish embryos compared with phenol red (PR)-injected controls. However, when macrophage numbers are depleted using an antisense oligonucleotide morpholino to the crucial macrophage transcription factor *irf-8* (Li et al., 2011), bacteria are able to proliferate in an uncontrolled manner and dominant active Hif-1αb stabilisation is powerless to decrease infection. Neutrophils, marked in green (using the *Tg(mpx:GFP)i114* transgenic zebrafish line; Renshaw et al., 2006), have emerging roles in granuloma formation and maintenance, but without macrophages present they cannot control infection alone. Images are from P.M.E. and A.H.M., unpublished observations.

Concurrent studies in zebrafish larvae shown that Hif-α signalling is important in *in vivo* infection, and is upregulated in leukocytes when zebrafish are challenged with lipopolysaccharide (a bacterial wall product) in hypoxia or with *M. marinum* in normoxia (Fig. 4; Elks et al., 2013; Liu et al., 2013). *Mycobacterium marinum* infection of zebrafish larvae is a well-established vertebrate tuberculosis model that has informed our understanding of the human disease (Berg and Ramakrishnan, 2012; Meijer and van der Vaart, 2014; Stoop et al., 2011). The temporal and spatial resolution of the live zebrafish *M. marinum* model has been used to demonstrate that Hif-α stabilisation in *M. marinum*-infected zebrafish macrophages is transient and rapidly downregulated, creating permissive conditions for bacterial growth (Elks et al., 2013). The modulation of Hif-α in zebrafish has demonstrated that this pathway intricately controls the production of neutrophil nitric oxide (NO). Overexpression of Hif-1α stimulated inducible nitric oxide synthase (Nos2a) to produce NO, an important antimicrobial mechanism of leukocytes during infection (Elks et al., 2013, 2014; see Box 1). Interestingly, Hif-1α and Hif-2α were found to have opposing functions on NO production. The stabilisation of Hif-1α primes neutrophils with increased NO levels, allowing the host to deal with infection better. Conversely, decreasing Hif-2α increases neutrophil NO levels (Elks et al., 2013). This complex regulatory signature of different Hif-α variants demonstrates the need for intact *in vivo* models, such as the zebrafish, with immune cells in their natural tissue environment in order to gain a proper understanding of the mechanisms involved and their precise effect on infection.

## HIF components as therapeutic targets: translational challenges and future prospects

Tissue hypoxia and HIF signalling have been implicated in many diseases, making the pathway an attractive target for therapeutic intervention. Future therapeutic strategies aimed at targeting HIF and hypoxia signalling to treat diseases such as cancer, inflammation and infection will depend on having a better understanding of the underlying biology of these conditions through the use of physiologically relevant disease models. Targeting such a fundamental, evolutionarily conserved pathway requires caution, and much remains to be understood about HIF regulation and its downstream effects in the whole-organism setting. There remain challenges to the translatability of hypoxia research into the clinic that newer models such as the zebrafish, in concert with more traditional cell-line and murine models, could help to address.

### HIF activation or inhibition

In complex disease syndromes, such as cancer, the inhibition of HIF might prove to be beneficial, whereas in others, such as infection, HIF activation has potential as a therapy. This ‘double-edged sword’ scenario is especially relevant *in vivo*, where modulating HIF in tumour cells may be beneficial , while having opposing, potentially unwanted effects on tumour-resident immune cells; *in vivo* zebrafish models have helped to identify mechanisms by which leukocytes are crucial effector cells in cancer (Feng et al., 2012). Therefore, experimental whole-organism models, such as the zebrafish, will facilitate the elucidation of the overall effect of HIF activation or inhibition both on the target cell type and on the organism as a whole.

Therapeutics that specifically target the HIF pathway have been difficult to identify and synthesise. The idea of downregulating HIF-α therapeutically has been around for a number of years, especially in the anaemia and cancer fields, and has been extensively reviewed elsewhere (Hu et al., 2013; Koh et al., 2010; Maxwell, 2005). A major drawback has been the substantial difficulty in designing HIF-specific inhibitors owing to the intracellular nature of the protein complex and the lack of active sites to which small molecular inhibitors are usually designed (Scheuermann et al., 2013). Current inhibitors of HIF signalling in early stages of clinical trials have been identified in screening studies and have indirect effects on HIF, via signalling components, including phosphoinositide 3-kinase (PI3K) and mammalian target of rapamycin (mTOR) (see Box 1), but few have yet to translate their promising *in vitro* potential into the clinic (Xia et al., 2012). As yet, there has been no HIF-specific inhibitor described in the literature. Although HIF-α proteins are difficult to target because of the lack of a traditional active site, there has been recent progress in the development of small molecules that target the Per-ARNT-Sim (PAS) protein interaction domain of HIF-2α, but not of HIF-1α, important in the assembly of the HIF complex (Scheuermann et al., 2013). This is because HIF-2α has a larger cavity in the PAS domain than does HIF-1α, providing access to water or small molecular antagonists (Scheuermann et al., 2009). Although these drugs are potentially exciting compounds that might enable us to dissect the roles of HIF-1α versus HIF-2α, their activity *in vivo* has yet to be elucidated. They will also be likely to produce off-target effects, because many proteins contain similar PAS domains. A second promising method to inhibit HIF-2α is the use of drugs that promote binding of intracellular iron regulatory protein 1 (IRP1; see Box 1) to the promoter of *HIF-2α* (but not *HIF-1α*) mRNA, repressing translation (Zimmer et al., 2008). The potential *in vivo* effectiveness of this strategy was recently demonstrated in the zebrafish *vhl* knockout model, where treatment with this class of drugs improved the disease phenotype (Metelo et al., 2015).

The prospect of activating and stabilising HIF-α as a therapeutic has had more success, in terms of both the number of pharmaceuticals that have been identified and the current status of translating these into clinical trials (Harten et al., 2010). The majority of these compounds target the regulatory hydroxylase enzymes, PHD and FIH, and include hydroxylase inhibitors, such as DMOG, FG-4497 and JNJ1935 (Barrett et al., 2011; Robinson et al., 2008). Although these drugs enhance HIF-α stability and activity, all are pan-hydroxylase inhibitors, so not only do they have effects on PHD-1, -2 and -3, but they also inhibit other hydroxylase families, such as collagen hydroxylases (Rose et al., 2011). Nonetheless, a series of hydroxylase inhibitors are in phase 2 and phase 3 clinical trials to treat anaemia in chronic kidney disease, paving the way for these drugs to be tested in other diseases.

Animal models of disease have an important place in the identification of new HIF-modulating compounds and in assessment of their toxicity and effectiveness *in vivo*. The zebrafish embryo represents a small, cost-effective, *in vivo* system, in which drugs can be screened in a 96-well format, allowing medium-high throughput screening to identify drugs that target HIF-α. Drug screening in zebrafish embryos has proved to be a successful approach to identify regulators of physiological or disease-related processes, including regulators of haematopoietic stem cell production and of reverse migration of neutrophils away from a site of inflammation (Kaufman et al., 2009; Robertson et al., 2014a), but has yet to be used to identify HIF modulators in disease settings. Small vertebrate disease models would allow the effect of a drug on a specific mechanism to be investigated in a whole-organism setting. The use of zebrafish in this way would allow off-target effects and toxicity to be studied in a simple assay. These *in vivo* assays will help to address the challenge of identifying novel Hif-modulating therapeutics in specific disease settings.

### HIF-1α, HIF-2α or HIF-3α

The regulation of the hypoxic response is closely controlled by the transcription and post-translational stabilisation of HIF-1α, HIF-2α and HIF-3α to produce the overall HIF-signalling effect (Keith et al., 2012). The best-understood HIF-α isoform in many disease settings is HIF-1α (Semenza, 2010). However, the role of HIF-2α has been implicated in diseases such as repetitive kidney cancer for more than a decade (Kondo et al., 2003; Zimmer et al., 2004). The differential roles of HIF-1α, HIF-2α and HIF-3α in disease are not well understood, at least in part because either physical hypoxia or hydroxylase inhibition is widely used as a stimulus in studies of HIF signalling. These methods stabilise all HIF-α variants, whereas in disease situations variant-specific stabilisation might occur in a spatial and temporal manner. Although there are well-characterised targets of HIF-α signalling [for example, erythropoietin, VEGF, PHD3 and nitric oxide synthase (NOS); see Box 1], there are potentially >1000 other direct and secondary targets of this pathway, many of which will be HIF-α-variant and cell-type specific (D'Angelo et al., 2003; Greenald et al., 2015; Liu et al., 1995; Palmer et al., 1998; Wang and Semenza, 1993). The zebrafish has well-conserved Hif-1α, Hif-2α and Hif-3α variants and given its genetic tractability and the range of diseases that can be modelled, it offers an opportunity to understand the interplay between HIF-α variants during disease in real time. Recent data from zebrafish have demonstrated that Hif-1α and Hif-2α have opposing effects on the production of NO by leukocytes (Elks et al., 2013). This is one example of many potential differential responses of HIF-α variants during disease. The ease with which the zebrafish can be genetically manipulated has increased our knowledge of the lesser-known Hif-3α variant, allowing investigation of its multiple splice variants in the setting of a simple organism, opening up unexplored possibilities of investigating the role of Hif-3α in disease settings (Kajimura et al., 2006; Zhang et al., 2014). With increased use of deep-sequencing techniques from a limited starting material, the identification of specific Hif-α variant targets during disease, in a cell-type-specific manner, is now a technical possibility in zebrafish models of disease (Rougeot et al., 2014). Understanding these finely balanced mechanisms will be important for the ultimate development of successful HIF-based therapeutics.

### Targeting HIF in a cell-type-specific manner

Exciting advances in drug-delivery technology mean that any detrimental off-target or unwanted effects caused by HIF-modulating therapeutics could be avoided by the direct delivery of a drug to the effector cell type of choice. In humans, one of the most advanced organ-specific drug-delivery technologies is the targeting of the liver using liposomes (see Box 1). Liposomes accumulate at high levels in the liver when administered systemically, potentially as a result of the liver vasculature having many dead ends (Lorenzer et al., 2015; Nair et al., 2014). There has been progress in knocking down HIF signalling using a small interfering (si)RNA against HIF-1β in hepatic cell lines and, although this has yet to be transferred into *in vivo* systems, it remains an exciting therapeutic possibility in human disease, using liposome-delivery technology (Choi et al., 2014).

Drug-delivery technologies have recently been successfully adapted in *in vivo* zebrafish models, using liposomes and synthetic polymersomes (a class of artificial vesicles; see Box 1) to deliver cargos to leukocytes (Fenaroli et al., 2014; Robertson et al., 2014b; Ruyra et al., 2014). These studies show a promising indication that HIF manipulation could be performed specifically in cell types of choice. However, more *in vivo* work must be done to develop this technology further and to expand the number of cell types that could be targeted for HIF manipulation.

## Conclusions

Hypoxia and HIF signalling play an integral role in disease processes, and *in vivo* studies have identified complex regulatory systems that involve an interplay between multiple HIF variants and different cell types that work together to elicit effects. Further studies of HIF signalling and its associated diseases in *in vivo* models are required to understand these complex processes and to identify potential therapeutic avenues. The zebrafish has emerged as an exciting and potentially translatable vertebrate model, in which to investigate the roles of HIFs in disease. Recent advances in genetic manipulation (CRISPR/Cas9) and microscopy [including lightsheet and single plane illumination microscopy (SPIM) techniques; see Box 1] further strengthen the zebrafish as a model (Kobitski et al., 2015). Although the investigation of Hif in zebrafish is relatively recent, important discoveries in these models mark out zebrafish as being an exciting future tool for understanding the complexities of HIF regulation during disease. Alongside cell and murine models, zebrafish will help in the search for therapeutic strategies to modulate HIF effectively in human disease.
